# Modification effect of sex and obesity on the correlation of *LEP* polymorphisms with leptin levels in Taiwanese obese women

**DOI:** 10.1002/mgg3.1113

**Published:** 2020-01-08

**Authors:** De‐Min Duan, Jing‐Yi Jhang, Semon Wu, Ming‐Sheng Teng, Lung‐An Hsu, Yu‐Lin Ko

**Affiliations:** ^1^ Division of Cardiology Department of Internal Medicine and The Cardiovascular Medical Center Taipei Tzu Chi Hospital Buddhist Tzu Chi Medical Foundation Taipei Taiwan; ^2^ Department of Life Science Chinese Culture University Taipei Taiwan; ^3^ Department of Research Taipei Tzu Chi Hospital Buddhist Tzu Chi Medical Foundation Taipei Taiwan; ^4^ The First Cardiovascular Division Department of Internal Medicine Chang Gung Memorial Hospital Taipei Taiwan; ^5^ Chang Gung University College of Medicine Taipei Taiwan; ^6^ Tzu Chi University College of Medicine Hualien Taiwan

**Keywords:** *LEP*, leptin, obesity, single‐nucleotide polymorphism

## Abstract

**Background:**

Obesity has become the main health issue in developed countries as it impacts life expectancy and increases mortality of cerebrovascular or cardiovascular diseases. The leptin is one of the adipokines which presents in the serum in proportion to the amount of adipose tissue and is translated from *LEP* gene. It involves in energy homeostasis, lipid and glucose metabolisms, modulation of immune systems, and thermogenesis. Many previous studies have revealed controversial results between *LEP* polymorphisms and leptin levels in different ages and ethnicities. Herein, we investigated the impacts of *LEP* polymorphism against leptin levels in Taiwanese subjects.

**Methods:**

In 599 Taiwanese subjects, excluding clinically overt systemic disease, age below 18 years old, and C‐reactive protein (CRP) level of above 10 mg/L, few of *LEP* polymorphisms were genotyped with TaqMan SNP genotyping assays, were further analyzed for association with leptin level in univariate and multivariate linear regression analyses with Bonferroni correction for multiple tests in stratified groups. The univariate and stepwise multivariate linear regression analyses were performed to determine the coefficient of determinant of *LEP* polymorphisms over leptin level.

**Results:**

Significant associations were found between *LEP* polymorphisms and leptin levels in obese women. Circulating leptin level was positively correlated with inflammatory, insulin resistance markers, and visceral obesity markers in all subjects. Furthermore, stratified and interaction analyses revealed that *LEP* polymorphisms, rs7799039 and rs2167270, were significantly associated with leptin levels in obese women—8%–10% of which could be explained by *LEP* polymorphisms.

**Conclusion:**

The *LEP* polymorphisms are independently associated with leptin levels in Taiwanese obese women. Further, the genetic determinants for leptin levels may be different between obese and nonobese, and in different sex individuals. The obesity status and female sex may exert modification effect on transcription of *LEP*, particularly in obese women.

## Graphical abstract

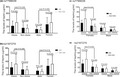

## 1 INTRODUCTION

Obesity, a prevalent condition in both developing and developed countries, results from both genetic and environmental factors. When body mass index (BMI) exceeds ≥20% of the ideal value—an indicator of obesity—life expectancy decreases. Leptin is an adipocyte‐derived hormone that suppresses food intake and increases energy expenditure by binding to and activating its specific receptor in the hypothalamus (Tartaglia et al., 1995; Woods & Stock, 1996). Leptin is released from white adipose tissue, and thus, leptin levels are positively correlated with body fat and body mass (Bribiescas & Hickey, 2006; Kelesidis, Kelesidis, Chou, & Mantzoros, 2010). Leptin levels increase with age and are higher in females than in males (Fulda et al., 2010). Several studies on the association of *LEP* (OMIM: 164160; GenBank: NC_000007.14) polymorphisms with BMI or obesity have been conducted. Although the *LEP* promoter polymorphism G‐2548A (rs7799039) has been studied intensively (De Silva et al., 1999; Duarte, Colagiuri, Palu, Wang, & Wilcken, 2003; Furusawa et al., 2011; Paracchini, Pedotti, & Taioli, 2005; Wang et al., 2006), the results for the associated single‐nucleotide polymorphism (SNP) and their alleles, which may indicate the roles of genetic and environmental factors in obesity, have been controversial. In a BMI‐adjusted meta‐analysis of genome‐wide association studies including both men and women, association of *LEP* SNPs reached genome‐wide significance in both sexes (*p* < 5 × 10^−8^) (Kilpeläinen et al., 2016). The *LEP* SNPs are associated with circulating leptin levels predominantly in obese subjects, especially in obese women and girls (Ma et al., 2009). Moreover, leptin levels increase with age and are higher in females than in males (Fulda et al., 2010); however, a study demonstrated a decline in leptin levels in women after menopause (Rosenbaum et al., 1996). In the present study, we investigated the genetic and environmental effects of *LEP* SNPs on obesity, obesity‐related metabolic traits, and leptin level.

## 2 METHODS

### 2.1 Ethical compliance

All individuals provided written informed consent. The study was approved by the Taipei Tzu Chi Hospital, Buddhist Tzu Chi Medical Foundation Institutional Review Board according to the guidelines of ICH‐GCP.

### 2.2 Study population

We recruited 599 Taiwanese individuals (men: *n* = 315, mean age = 46.1 ± 10.0 years; women: *n* = 284, mean age = 46.8 ± 9.9 years) during routine health examinations between October 2003 and September 2005 in the Chang Gung Memorial Hospital, Taiwan. Their self‐reported medical history and lifestyle characteristics were recorded; next, in their physical examination, we measured their height, weight, waist and hip circumferences, and blood pressure in the sitting position after 15 min of rest. Fasting blood samples were obtained from each individual. Exclusion criteria included age <18 years; C‐reactive protein (CRP) level >10 mg/L; and history of myocardial infarction, stroke, or transient ischemic attack, cancer, and current renal or liver disease. Obesity was defined as BMI ≥25 kg/m^2^, according to the Asian criteria (WHO Expert Consultation, 2004). Current smokers were defined as those who smoked at least one cigarette per day at the time of the survey. The clinical characteristics and biometrics of the study population are summarized in Table 1.

**Table 1:** Clinical and biochemical characteristics of participants

	Obese		Nonobese		*p* value
	Women	Men	Women	Men	—
Number, *n* (%)	86	147	198	168	
Age (years)	49.66 ± 9.41	45.41 ± 9.59	45.55 ± 9.88	45.65 ± 10.3	.005
Body mass index (kg/m^2^)	27.90 ± 2.60	27.52 ± 2.23	21.65 ± 1.90	22.71 ± 1.75	2.00 × 10^–130^
Waist–hip ratio	0.88 ± 0.07	0.89 ± 0.04	0.83 ± 0.06	0.87 ± 0.05	1.46 × 10^–26^
Waist circumference (cm)	91.81 ± 9.75	93.23 ± 6.16	77.61 ± 7.08	83.45 ± 5.59	8.38 × 10^–81^
Systolic BP (mm Hg)	120.22 ± 18.19	118.76 ± 16.45	111.18 ± 18.45	114.05 ± 15.89	.00001
Diastolic BP (mm Hg)	77.33 ± 10.16	79.80 ± 10.24	72.19 ± 9.97	76.38 ± 10.05	1.47 × 10^–10^
Glucose AC (mg/dl)	100.94 ± 26.64	99.58 ± 21.05	91.69 ± 16.19	99.63 ± 30.16	.001
Insulin	10.42 ± 3.85	12.06 ± 6.78	7.72 ± 3.38	7.81 ± 3.05	5.91 × 10^–21^
HOMA‐IR index	2.68 ± 1.59	3.01 ± 1.93	1.75 ± 0.83	1.92 ± 1.06	7.06 × 10^–24^
QUICKI	0.33 ± 0.02	0.33 ± 0.025	0.35 ± 0.02	0.35 ± 0.02	1.83 × 10^–23^
Total cholesterol (mg/dl)	197.46 ± 35.76	204.68 ± 36.77	196.19 ± 35.77	197.16 ± 36.73	.149
HDL cholesterol (mg/dl)	55.17 ± 12.12	48.42 ± 10.32	63.93 ± 14.24	51.01 ± 13.05	2.38 × 10^–23^
LDL cholesterol (mg/dl)	115.53 ± 30.92	120.31 ± 32.72	112.29 ± 31.67	116.58 ± 34.80	.161
Triglyceride (mg/dl)	132.69 ± 63.72	192.34 ± 150.28	100.63 ± 57.84	154.95 ± 142.79	6.68 × 10^–12^
Creatinine (mg/dl)	0.90 ± 0.76	1.13 ± 0.27	0.80 ± 0.12	1.11 ± 0.55	4.83 × 10^–11^
Current smokers (%)	3 (3.5%)	58 (39.5%)	8 (4%)	48 (28.6%)	.079
C‐reactive protein (mg/L)	1.58 ± 1.37	1.20 ± 1.30	0.84 ± 1.34	1.01 ± 1.45	3.38 × 10^–10^
Leptin (g/L)	44.92 ± 26.49	15.15 ± 9.5	21.81 ± 17.11	7.31 ± 5.08	2.14 × 10^–93^

Note

Data are presented as mean ± standard deviation or percentage as appropriate. Obesity was defined as BMI ≥25 kg/m^2^ according to the Asian criteria (WHO Expert Consultation, 2004).

Abbreviations: BP, blood pressure; HDL, high‐density lipoprotein; HOMA‐IR, homeostatic model assessment of insulin resistance; LDL, low‐density lipoprotein; QUICKI, quantitative insulin sensitivity check index.

### 2.3 Genomic DNA extraction and genotyping

Genomic DNA was extracted as reported previously (Teng et al., 2013). Two SNPs around *LEP*, rs7799039 (HGVS nomenclature: NC_000007.13:g.127878783A>G) and rs2167270 (HGVS nomenclature: NM_000230.2:c.‐39G>A), were selected. Genotyping was performed using TaqMan SNP with genotyping assays (Applied Biosystems).

### 2.4 Laboratory examinations and assays

The laboratory examinations and assays were performed as described below. Fasting plasma glucose levels and lipid profiles were obtained, and the homeostatic model assessment of insulin resistance (HOMA‐IR) index and quantitative insulin sensitivity check index (QUICKI) were calculated as reported previously (Wu et al., 2014). Most markers, including serum CRP, soluble E‐selectin (sE‐selectin), soluble P‐selectin (sP‐selectin), and adiponectin, were measured using a sandwich enzyme‐linked immunosorbent assay (ELISA) developed in‐house. All in‐house kits demonstrated good correlation compared with commercially available ELISA kits. Circulating lipocalin‐2 and sP‐selectin were measured using commercially available ELISA kits from R&D Systems. Furthermore, serum insulin levels were measured using an immunoradiometric assay kit from BioSource. ELISA and genotyping were performed by laboratory personnel blinded to the clinical status of the participants.

### 2.5 Statistical analysis

The statistical analysis was performed on IBM SPSS Statistics (version 22; IBM) unless otherwise specified. The independent samples *t* test was performed for categorical variables. The continuous variables, expressed as mean ± standard deviation, were tested using one‐way analysis of variance (ANOVA) with Bonferroni correction for multiple tests. Tests of normality were conducted for all quantitative traits. Moreover, the HOMA‐IR index and leptin, insulin, triglyceride (TG), sE‐selectin, sP‐selectin, adiponectin, and CRP levels were logarithmically transformed before statistical analysis to adhere to a normality assumption. A *p* of < .05 through a two‐sided test was considered statistically significant. Linear regression coefficients with 95% confidence intervals were calculated for leptin levels and the predicted confounders. Allelic frequencies for each SNP were estimated through gene counting, and the polymorphism distribution was tested for Hardy–Weinberg equilibrium using the Chi‐square test. The stratified association analysis according to sex and obesity was performed using one‐way ANOVA with Bonferroni correction for multiple tests in additive and dominant genetic models. Furthermore, we investigated the sex‐ and obesity‐specific effects of leptin level variants. The resultant significant polymorphisms were included in interaction analysis using a linear regression model of SVS Win32 (version 7.3.1; Golden Helix) to determine the impact of dependent variables. The univariate and stepwise multivariate linear regression analyses were performed to detect the coefficients of determination of genetic and traditional predictors against leptin level in both all subjects and obese women groups. The statistical power of >0.8 was obtained in the univariate analyses of *LEP* polymorphisms and leptin levels in the dominant model using alpha of 0.05. Linkage disequilibrium (LD) was analyzed by *D′* and *r* ^2^ measures.

## 3 RESULTS

### 3.1 Clinical and biochemical characteristics

The participants were stratified into four groups according to sex and obesity status; the demographic features, clinical profiles, and levels of biomarkers are listed in Table 1. The obese women were older and demonstrated significantly higher metabolic traits, including BMI, waist circumference, waist‐to‐hip ratio (WHR), systolic and diastolic blood pressures, and leptin level. The obese men and women had significantly higher HOMA‐IR index, insulin, and CRP levels but lower QUICKI levels than did their nonobese counterparts.

### 3.2 Correlations between leptin levels and clinical parameters and biomarker levels

In the general population, circulating leptin level was significantly and positively correlated with BMI, waist circumference, systolic blood pressure, insulin, HOMA‐IR index, and CRP levels but negatively with QUICKI in all individuals. Similar correlations, except those for waist circumference, were found in obese women. In addition, diastolic blood pressure and sE‐selectin level were significantly and positively correlated with leptin level in obese women (Table 2).

**Table 2:** Association between leptin levels and measurable risk factors in Taiwanese individuals

	All subjects		Obese women	
	r		r	*p* value
Age	0.064	0.060	−0.031	.386
Body mass index	0.350	2.72 × 10^–18^	0.323	.001
Waist circumference	0.205	3.85 × 10^–7^	0.044	.345
Waist–hip ratio	−0.006	0.437	−0.113	.152
Systolic BP	0.125	0.001	0.249	.011
Diastolic BP	0.039	0.174	0.327	.001
Fasting plasma glucose	−0.058	0.082	−0.164	.066
Fasting serum insulin	0.371	1.92 × 10^–20^	0.350	.0005
HOMA‐IR index	0.311	1.39 × 10^–14^	0.201	.033
QUICKI	−0.320	2.11 × 10^–15^	−0.219	.022
Total cholesterol	−0.008	0.418	−0.055	.307
LDL cholesterol	−0.045	0.137	−0.066	.272
HDL cholesterol	0.095	0.011	0.098	.185
Triglyceride	0.013	0.375	−0.083	.223
CRP	0.205	3.61 × 10^–7^	0.261	.008
sE‐selectin	0.051	0.107	0.237	.014
sP‐selectin	−0.093	0.012	−0.128	.122
Lipocalin2	0.031	0.225	0.094	.195
Adiponectin	0.078	0.029	0.086	.217

### 3.3 Associations of *LEP* polymorphisms with respective circulation levels in different sexes and obesity status

The allele frequencies at all polymorphisms satisfied the Hardy–Weinberg equilibrium and agreed closely with the allele frequencies described at these polymorphisms in the study population (Table 3). The association analyses in additive and dominant models were adjusted for age and smoking status. In the additive model, minor alleles of rs7799039 and rs2167270 were associated with higher leptin levels in obese individuals (*p* = .003 and .001, respectively; Table 4) and women (*p* = .025 and .031, respectively). This association also persisted in the dominant model for rs7799039 and rs2167270 in obese individuals (*p* = .003 and .028, respectively) and women (*p* = .015 and .040, respectively; Table 4). We stratified the individuals into four groups to analyze the associations between *LEP* polymorphisms and leptin levels with respect to sex and obesity status. In the dominant model, the associations between *LEP* polymorphisms and leptin level were significant only in obese women (rs7799039; *p* = .008; rs2167270, *p* = .006; Table S1).

**Table 3:** The allele frequency distributions of *LEP* SNPs and Hardy–Weinberg equilibrium (HWE) tests

SNP	Chr		Position		Minor allele	Allele frequency (CHS)	HWE *p* value
rs7799039		7	127,878,783	intergenic variant	G	0.286	.580
rs2167270		7	127,881,349	5′ UTR	A	0.219	.254

Note

*p* adjusted for sex, age, BMI, and smoking status.

Abbreviations: Allele frequency, data from 1,000 Genome project Phase 3; Chr, chromosome; CHS, southern Han in China; *LEP* GenBank Ref Seq, NC_000007.14.

**Table 4:** Associations of *LEP* SNPs with leptin levels in relation to sex and obesity status

Genotype	MM	Mm	mm	β (95% CI)	*p*	MM	Mm+mm	*p*
rs7799039	AA	AG	GG			AA	AG + GG	
Total	17.56 ± 16.62 (301)	21.41 ± 21.88 (251)	23.15 ± 19.36 (37)	0.051 (0.001–0.102)	.045	17.56 ± 16.62 (301)	21.64 ± 21.55 (288)	.115
Obese	20.61 ± 13.04 (117)	32.07 ± 29.19 (100)	31.31 ± 26.97 (14)	0.103 (0.035–0.170)	.003	20.61 ± 13.04 (117)	31.98 ± 28.82 (114)	.003
Nonobese	15.62 ± 18.32 (184)	14.36 ± 10.48 (151)	18.19 ± 10.75 (23)	0.015 (−0.049 to 0.078)	.652	15.62 ± 18.32 (184)	14.86 ± 10.56 (174)	.849
Male	10.99 ± 7.41 (168)	11.00 ± 10.05 (127)	10.62 ± 6.30 (14)	−0.023 (−0.089 to 0.044)	.505	10.99 ± 7.41 (168)	10.96 ± 9.73 (141)	.439
Female	25.86 ± 20.84 (133)	32.08 ± 25.36 (124)	30.79 ± 20.72 (23)	0.059 (0.007–0.111)	.025	25.86 ± 20.84 (133)	31.88 ± 24.63 (147)	.028
rs2167270	GG	GA	AA			GG	GA + AA	
Total	17.91 ± 17.09 (331)	21.45 ± 21.71 (229)	24.91 ± 22.87 (23)	0.041 (−0.013 to 0.095)	.137	17.91 ± 17.09 (331)	21.77 ± 21.79 (252)	.256
Obese	21.61 ± 15.30 (133)	32.02 ± 29.07 (90)	37.61 ± 33.91 (8)	0.096 (0.022–0.169)	.011	21.61 ± 15.30 (133)	32.47 ± 29.34 (98)	.015
Nonobese	15.43 ± 17.81 (198)	14.61 ± 10.66 (139)	18.14 ± 10.29 (15)	0.010 (−0.057 to 0.078)	.761	15.43 ± 17.81 (198)	14.95 ± 10.64 (154)	.912
Male	11.52 ± 9.30 (186)	10.25 ± 7.23 (112)	10.63 ± 7.42 (8)	−0.052 (−0.123–0.020)	.157	11.52 ± 9.30 (186)	10.27 ± 7.21 (120)	.139
Female	26.12 ± 20.92 (145)	32.18 ± 25.28 (117)	32.53 ± 24.81 (15)	0.061 (0.006–0.116)	.031	26.12 ± 20.92 (145)	32.22 ± 25.13 (132)	.040

Note

Leptin levels, Means ± *SD* (*N*). *p* adjusted for age and smoking status.

Abbreviations: m, minor allele; M, major allele.

### 3.4 Interaction analysis

**Figure 1:**
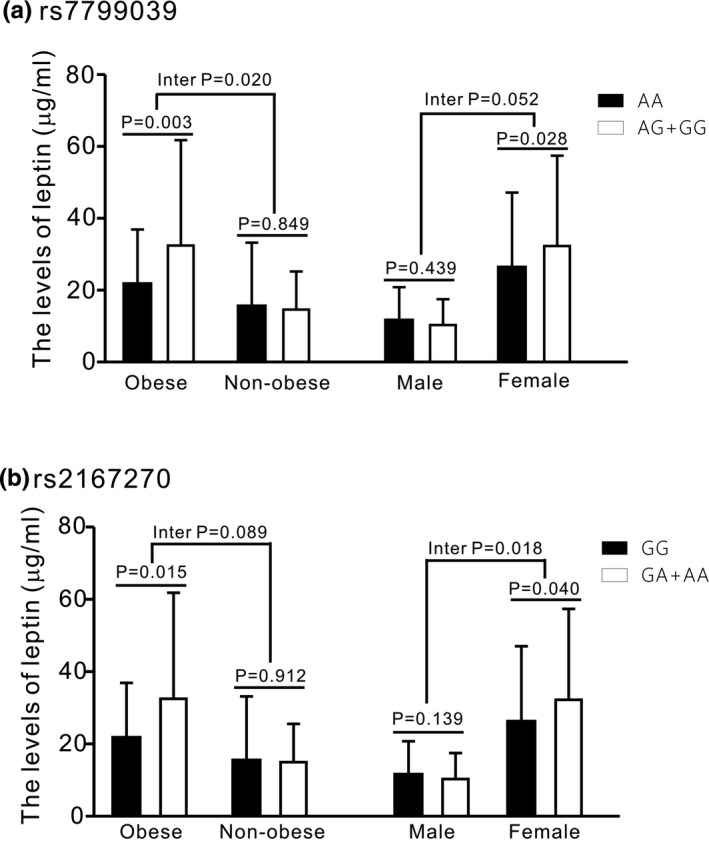
Association and interaction analysis between *LEP* polymorphisms and leptin levels in different groups (*P* adjusted for age and smoking status)

**Figure 2:**
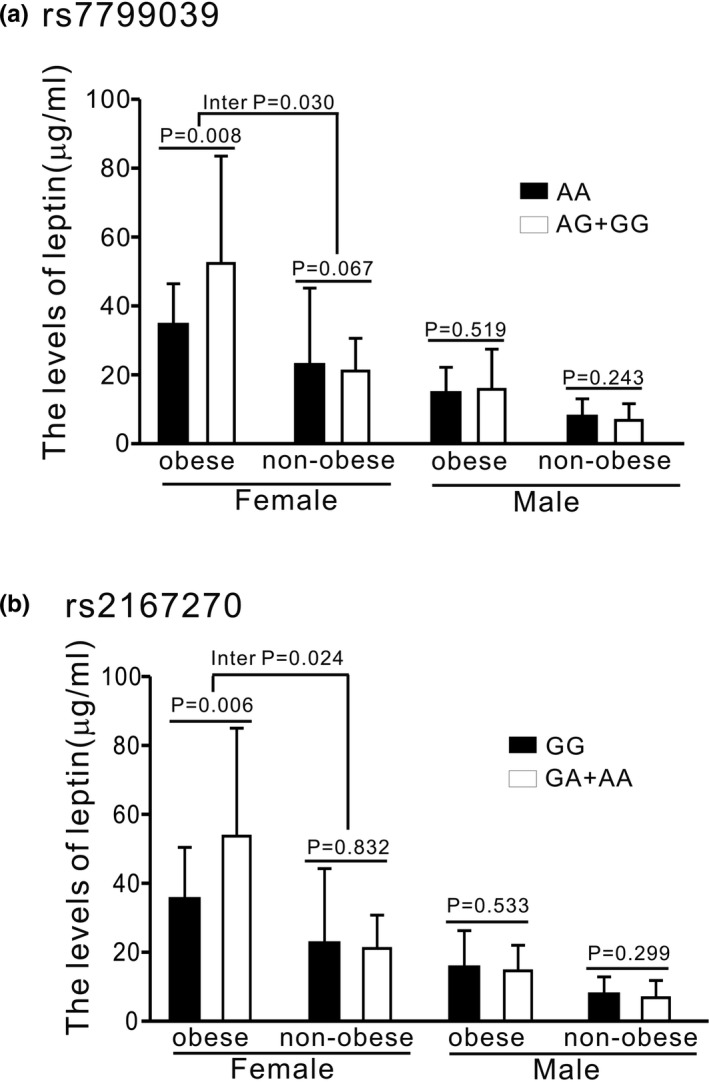
Association and interaction analysis between *LEP* polymorphisms and leptin levels in different groups (*P* adjusted for age and smoking status)

Regarding the association of *LEP* polymorphisms with leptin levels, the interaction with obesity existed in the dominant model of rs7799039 in obese individuals and that of rs2167270 in women (interaction *p* = .020 and .018, respectively; Figure 1a,b). Nevertheless, the interaction with obesity existed in the dominant model of rs7799039 and rs2167270 in the subgroup analysis for obese women (interaction *p* = .030 and .024, respectively; Figure 2a,b). In addition, strong LD was detected between rs7799039 and rs2167270 (*r* ^2^ = .728; *D′* = 0.999).

### 3.5 Coefficient of determinant of polymorphisms over leptin level

Both the univariate and stepwise multivariate linear regression revealed significant determinants of dominant models of both rs7799039 and rs2167270 over substantial variation in circulating leptin levels exclusively in obese women (*p* = .003 and .002 for univariate analyses and *p* = .005 and .016 for multivariate analyses, respectively), 8%–10% of which could be explained by *LEP* polymorphisms (Table 5). In contrast, traditional predictors, not *LEP* polymorphisms, conferred significant determinants in all subjects group (Table S2).

**Table 5:** Coefficient of determination of genetic and traditional predictors over leptin levels in obese women

Predictors	Univariate analysis			Stepwise multivariate analysis					
	Beta (SE)	R^2^	*p* value	Beta (SE)	R^2^	*p* value*	Beta (SE)	R^2^	*p* value*
rs7799039 AG + GG	0.138 (0.308)	0.095	.003	0.118 (0.041)	0.092	.005	—	—	—
rs2167270 GA + AA	0.142 (0.319)	0.102	.002	—	—	—	0.104 (0.042)	0.080	.016
Insulin	0.514 (0.145)	0.128	.0006	0.505 (0.133)	0.152	.0002	0.482 (0.135)	0.152	.0006
DBP	1.209 (0.397)	0.099	.003	0.836 (0.360)	0.047	.360	0.795 (0.369)	0.042	.034
BMI	1.937 (0.596)	0.111	.0016	—	—	—	—	—	—
SBP	0.811 (0.361)	0.056	.027	—	—	—	—	—	—
CRP	0.105 (0.052)	0.045	.049	—	—	—	—	—	—
sE‐selectin	0.262 (0.119)	0.054	.031	—	—	—	—	—	—

* adjusted for BMI, systolic and diastolic pressures, insulin levels, CRP, and sE‐selectin level.

## 4 DISCUSSION

Leptin is an adipokine derived from adipocytes in the white adipose tissue; its serum levels are in general in proportion with the amount of adipose tissue. It is translated from *LEP* and is involved in energy homeostasis, lipid and glucose metabolisms, immune system modulation, and thermogenesis. In the present study, leptin levels were significantly and positively correlated with BMI, waist circumference, high blood pressure, and insulin resistance and inflammatory marker levels. The highest leptin level and a similar correlation were noted in obese women.

Several studies have demonstrated the correlation of leptin with metabolic syndrome, including visceral obesity, hypertension, insulin resistance, and dyslipidemia. In Taiwanese school‐aged children, high leptin levels were correlated with high body weight, BMI, WHR, and blood pressure (Chu, Wang, & Shieh, 2001). The higher leptin level increased the risk of metabolic syndrome in Taiwanese individuals (Li et al., 2011). In the Copenhagen City Heart Study, leptin was significantly associated with new‐onset hypertension with an odds ratio of 1.28 (Asferg et al., 2010). In addition, the leptin level is positively correlated with blood pressure independent of body weight (Agata et al., 1997). A similar result was reported in a US adult population after adjustments for age, sex, and BMI (Shankar & Xiao, 2010). In a Polish study, postmenopausal hypertensive women had the highest leptin level among their counterparts, independent of their BMI (Olszanecka, Posnik‐Urbanska, Kawecka‐Jaszcz, Czarnecka, & Fedak, 2010). During pregnancy, leptin‐treated Sprague Dawley rats showed significantly lower angiotensin‐converting enzyme 2 levels, which cleaved angiotensin I and II into angiotensin 1–9 and 1–7, respectively; both of these products have vasodilatory, hypotensive effects, countering the effects of angiotensin II, finally leading to increase in blood pressure (Ibrahim, Froemming, Omar, & Singh, 2014). The chronic elevation of plasma leptin levels increases renal Na^+^, K^+^‐ATPase activity and subsequently disrupts natriuresis, elevating blood pressure (Beltowski, 2010). Our results showed that increased leptin levels may be associated with insulin resistance because leptin levels were positively correlated with fasting serum insulin levels and HOMA‐IR index and negatively with QUICKI. In the Brazilian study, higher leptin levels were positively correlated with insulin resistance (HOMA‐IR index) in children and adolescents (Gonzaga, Medeiros, de Carvalho, & Alves, 2014). In addition, the fasting leptin level is negatively associated with insulin sensitivity in women with a previous history of gestational diabetes mellitus (Madarász et al., 2009). Leptin also potentially exerts its function through an inflammatory pathway. CRP levels are positively correlated with leptin levels in women with preeclampsia (Molvarec et al., 2011).

*LEP*, a candidate gene of obesity mapped in chromosome 7q31.3, comprises three exons spanning approximately 20 kb and encodes a 16‐kDa protein, leptin. In our study, after adjustments for age and smoking status, both *LEP* SNPs rs7799039 and rs2167270 were significantly associated with leptin levels in obese individuals and women in either the dominant or additive model. These significant associations persisted in the dominant models of *LEP* polymorphisms in obese women. Obesity status and female sex interact with *LEP* polymorphisms, leading to higher leptin level either in all individuals or in obese women. Although several studies have reported controversial results regarding the associations of rs7799039 and rs2167270 with leptin levels, however, most results were consistent with our current results. In the association study of three Tunisian consanguineous families, the minor allele of *LEP* rs2167270 (A), but not rs7799039, was positively and significantly associated with leptin level (Fourati et al., 2013). The rs2167270 was predicted to modify the transcription factor binding sites (Jiang et al., 2004). This SNP may affect gene expression at a transcriptional level, leading to disrupted leptin production. However, the rs7799039 GA/AA polymorphisms were also not associated with leptin level in Egyptian individuals (Motawi, Salman, Shaker, & Abdelhamid, 2015). Different polymorphism frequencies in different populations may affect leptin levels. In addition, the association between rs7799039 and either plasma leptin level or gestational diabetes mellitus risk was not found in Chinese population (Yang et al., 2016). A Polish study reported no significant association of rs7799039 and rs12672770 with either relative mRNA level in subcutaneous adipose tissue or serum leptin levels in children and adolescents (Cieslak et al., 2012). The individuals who were exposed to prenatal famine in the Netherlands were investigated, the study revealed that *LEP* methylation was higher in the experimental groups, this could be explained by *LEP* methylation differences, which may depend on sex and gestational timing (Tobi et al., 2009). The correlation of *LEP* rs2167270 polymorphisms with increased BMI and waist circumference in women has been previously reported (Fourati et al., 2013; Friedlander et al., 2010). In our present study, rs1267270 was associated with BMI in additive or dominant models in obese women (*p* = .048 and *p* = .019, respectively).

In the interaction analysis, obesity status and female sex had a significant interactive effect on *LEP* polymorphisms related to leptin level in rs7799039 and rs2167270, respectively. When we compared the obese and nonobese women, we found that obesity status had an interaction effect on leptin level. Fat distribution, particularly subcutaneous abdominal fat as a determinant of leptin level, contributes to the variability in leptin level in obese individuals (Minocci et al., 2000). The sex difference in circulating leptin levels can be due to at least two mechanisms: higher proportion of adipose tissue and increased production rate of leptin per unit mass of adipose tissue (Hellström, Wahrenberg, Hruska, Reynisdottir, & Arner, 2000). Thus, obesity status may modify leptin levels directly or indirectly through gene transcription. rs7799039 (–2548G/A) influences leptin expression, possibly at the transcriptional level, and therefore, also adipose secretion levels of the hormone (Hoffstedt, Eriksson, Mottagui‐Tabar, & Arner, 2002). The polymorphism located in the promoter region of the leptin gene appears to be associated with large variations in leptin level in obese humans (given their wide range of adiposity) (Le Stunff, Le Bihan, Schork, & Bougnères, 2000). In univariate and multivariate analyses, the significant coefficient of determination of *LEP* polymorphisms, dominant models of both rs7799039 and rs2167270, over substantial variation in circulating leptin levels in obese women was observed, 8%–10% of which was explained by genetic predictors. As abovementioned, we supposed that *LEP* polymorphisms are closely correlated with leptin levels exclusively in obese women, and obesity status and female sex may exert modifying effects on *LEP* transcription.

## 5 CONCLUSION

The *LEP* polymorphisms are independently associated with leptin levels in Taiwanese obese women. Furthermore, the genetic determinants for leptin levels may differ between obese and nonobese individuals and between sexes. Obesity status and female sex may exert modifying effects on *LEP* transcription, particularly in obese women.

## CONFLICT OF INTEREST

There are no conflict to declare.

## AUTHOR CONTRIBUTIONS

DMD and JYJ participated in genotyping, performed statistical analysis, and drafted the manuscript. MST prepared the DNA samples and participated in genotyping. LAH participated in sample collection and prepared the DNA samples. DMD and JYJ performed and corrected statistical analysis. YLK and SW supervised the study and revised the manuscript. All authors read and approved the final version of the manuscript.

## Data Availability

The data that support the findings of this study are available on request from the corresponding author. The data are not publicly available due to privacy or ethical restrictions.
