# Supplementary material for: Modification effect of sex and obesity on the correlation of LEP polymorphisms with leptin levels in Taiwanese obese women
Source: Mol Genet Genomic Med. 2020 Jan 8;8(3):e1113. doi: 10.1002/mgg3.1113 (PMC7057123; doi:10.1002/mgg3.1113)
Supplement: Supplementary file 1 [file MGG3-8-e1113-s001.docx]

|  | male | Obese | | Non-obese | | Interaction |
| --- | --- | --- | --- | --- | --- | --- |
| SNP number | genotypes | Leptin levels  Means ± SD (N) | *P* value | Leptin levels  Means ± SD (N) | *P* value | *P* value |
| LEP  rs7799039 | AA  AG  GG  AA  AG+GG | 14.50±7.74(81)  15.86±12.21(58)  16.40±3.97(6)  14.50±7.74(81)  15.91±11.67(64) | 0.384  0.519 | 7.71±5.33(87)  6.91±5.00(69)  6.28±3.49(8)  7.71±5.33(87)  6.85±4.85(77) | 0.254  0.243 | 0.139  0.170 |
| LEP  rs2167270 | GG  GA  AA  GG  GA+AA | 15.49±10.79(92)  14.29±7.45(50)  17.73±5.69(3)  15.49±10.79(92)  14.48±7.36(53) | 0.733  0.533 | 7.63±5.23(94)  6.99±5.13(62)  6.36±4.42(5)  7.63±5.23(94)  6.94±5.05(67) | 0.285  0.299 | 0.547  0.671 |
|  | female | Obese | | Non-obese | | Interaction |
| SNP number | genotypes | Leptin levels  Means ± SD (N) | *P* value | Leptin levels  Means ± SD (N) | *P* value | *P* value |
| LEP  rs7799039 | AA  AG  GG  AA  AG+GG | 34.34±12.11(36)  54.45±31.08(42)  42.50±31.72(8)  34.34±12.11(36)  52.54±31.17(50) | 0.080  0.008 | 22.71±22.51(97)  20.63±9.77(82)  24.54±7.15(15)  22.71±22.51(97)  21.23±9.49(97) | 0.315  0.670 | 0.261  0.030 |
| LEP  rs2167270 | GG  GA  AA  GG  GA+AA | 35.32±15.12(41)  54.18±30.85(40)  49.53±39.01(5)  35.32±15.12(41)  53.67±31.37(45) | 0.020  0.006 | 22.49±21.82(104)  20.75±9.99(77)  24.03±6.35(10)  22.49±21.82(104)  21.13±9.67(154) | 0.552  0.832 | 0.088  0.024 |

Supplemental Table 1. Changes in associations between *LEP* polymorphisms with leptin levels with obesity status and sex

*P* adjusted for age and smoking status.

Supplemental table 2. Coefficient of determination of genetic and traditional predictors over leptin levels in all subjects

| Predictors | Univariate analysis | | | Stepwise multivariate analysis | | | | | |
| --- | --- | --- | --- | --- | --- | --- | --- | --- | --- |
|  | Beta (SE) | R^2^ | P value | Beta (SE) | R^2^ | P value* | Beta (SE) | R^2^ | P value* |
| rs7799039  AG+GG | 0.051 (0.031) | 0.004 | 0.109 | 0.030 (0.027) | - | 0.258 | - | - | - |
| rs2167270  GA+AA | 0.041 (0.032) | 0.002 | 0.203 | - | - | - | 0.008 (0.027) | - | 0.765 |
| Insulin | 0.780 (0.076) | 0.147 | 1.48×10^-22^ | 0.684 (0.084) | 0.146 | 2.66×10^-15^ | 0.672 (0.084) | 0.146 | 1.02×10^-14^ |
| BMI | 2.32 (0.244) | 0.131 | 4.69×10^-20^ | 2.737 (0.368) | 0.043 | 4.17×10^-13^ | 2.743 (0.368) | 0.044 | 3.76×10^-13^ |
| HDL | 0.211 (0.142) | 0.004 | 0.137 | 0.633 (0.146) | 0.059‬ | 0.000017 | 0.639 (0.146) | 0.058 | 0.000016 |
| Adiponectin | 0.059 (0.053) | 0.002 | 0.273 | 0.240 (0.055) | 0.023‬ | 0.000019 | 0.230 (0.055) | 0.022‬ | 0.000041 |
| Waist circumference | 1.672 (0.319) | 0.043 | 2.35×10^-7^ | -1.366 (0.456) | 0.009 | 0.002 | -1.379 (0.458) | 0.009 | 0.002 |
| CRP | 0.163 (0.032) | 0.041 | 4.76×10^-7^ | 0.083 (0.030) | 0.009 | 0.007 | 0.088 (0.031) | 0.010 | 0.004 |
| HOMA-IR | 0.583 (0.069) | 0.105 | 3.64×10^-16^ |  |  |  |  |  |  |
| QUICKI | -5.174 (0.595) | 0.112 | 3.63×10^-17^ |  |  |  |  |  |  |
| SBP | 0.656 (0.243) | 0.012 | 0.007 |  |  |  |  |  |  |

-* adjusted for BMI, waist circumference, systolic blood pressure, insulin, HDL, CRP, sP-selectin and adiponectin levels
